# Sublethal insecticide exposure and inbreeding impose a developmental bottleneck but enhance crop-damaging traits in surviving Colorado potato beetles

**DOI:** 10.1098/rsos.261014

**Published:** 2026-11-04

**Authors:** Nijat Nariman, Himrekha Agarwal, Shuqing Xu

**Affiliations:** Institute of Organismic and Molecular Evolution (iomE), Johannes Gutenberg-University of Mainz, 55128 Mainz, Germany

**Keywords:** *Leptinotarsa decemlineata*, developmental success, fitness, feeding behaviour, locomotor activity, phenotypic plasticity, reproduction, sex-specific response

## Abstract

Intensive agricultural insecticide use often fails to eradicate pest populations completely, leaving small remnant populations exposed to persistent sublethal residues and potentially elevated inbreeding. How such remnant populations persist remains poorly understood. Here, we examined how inbreeding and sublethal imidacloprid exposure shape survival and post-emergence performance in the Colorado potato beetle (*Leptinotarsa decemlineata*), a major agricultural pest. We compared four groups differing in breeding status and insecticide exposure history, and quantified emergence rate, locomotor activity, feeding behaviour and reproductive output. We found that despite the insecticide dosage being sublethal, both chemical exposure and inbreeding imposed severe developmental costs, reducing the adult emergence rate with no significant interaction effects. However, among the adults, we observed trait-specific phenotypic plasticity. Male locomotor activity was reduced by both stressors, while female activity remained stable. Notably, herbivory significantly increased under both inbreeding and sublethal insecticide exposure. Furthermore, while inbreeding severely reduced both egg production and hatching success, sublethal exposure to the insecticide surprisingly increased hatching success in surviving pairs. These results demonstrate that sublethal insecticide exposure and inbreeding can create a strong developmental bottleneck. Yet, surviving individuals can exhibit compensatory plasticity that maintains or even enhances their capacity for crop damage.

## 1. Introduction

With ongoing agricultural intensification worldwide, insecticide use is increasing, despite the well-documented capacity of many pest species to evolve resistance [1,2]. Intensive insecticide application may therefore not only suppress pest populations in the short term but also leave behind small remnant populations in treated fields, where persistent residues continue to expose developing offspring and immigrating individuals to sublethal stress. Such exposure can affect both fitness and behaviour (e.g. bed bug [3]; leaf beetle [4,5]; jumping spider [6]; damselfly [7]). These post-treatment bottlenecks may reshape populations through two distinct processes: first, by filtering development before adulthood, removing individuals unable to cope with genetic and chemical stress, and second, among those that do survive to adulthood, by favouring phenotypic plasticity that allows survivors to continue feeding, moving and reproducing despite continued stress. These processes act on different life stages and can occur separately or together; a population may show strong developmental filtering without notable survivor plasticity or vice versa. In agricultural systems, this distinction may be especially important because demographic reduction can increase the likelihood of mating among relatives while remnant populations remain exposed to persistent sublethal residues. However, it remains poorly understood how small remnant pest populations respond after insecticide-driven bottlenecks under these conditions [8].

Inbreeding depression—reduced fitness in the offspring produced by matings between genetically related individuals—is widespread in both animal and plant populations and is generally attributed to increased expression of recessive deleterious alleles and/or the loss of advantageous heterozygosity, which can impose substantial fitness costs [9]. Although an increasing body of work points to limited or absent inbreeding avoidance in natural populations [10], and some species even show a tendency to inbreed with genetically related partners (e.g. Mongolian gerbils [11] and naturally inbreeding social spider species [12]), inbreeding remains a potentially important constraint on the persistence of small remnant populations. This may be especially relevant for alien and invasive taxa, which often experience strong genetic bottlenecks and may sometimes withstand inbreeding depression or even derive benefits associated with inbreeding under certain conditions [13,14].

These dynamics are particularly pertinent in agricultural systems, where remnant pest populations can face both genetic and chemical stress at the same time. Inbreeding depression and sublethal insecticide exposure are each known to impair individual fitness and behaviour. Although inbreeding-by-stress interactions have been documented under thermal [15], nutritional [16] and some chemical stressors (i.e. cadmium, copper sulphate or methanol; [17,18]), evidence for how inbreeding and insecticide exposure shape survival and survivor phenotypes in pest populations remains limited. After intensive insecticide application, small remnant populations may experience elevated inbreeding while continuing to develop under persistent sublethal residues, yet the consequences of this combination for persistence in managed agroecosystems remain unclear [19].

Inbreeding and sublethal chemical exposure may affect females and males differently, reflecting sex-specific life-history traits, reproductive investment and physiological tolerance [20,21]. Both inbreeding depression and sublethal insecticide effects can be sex-specific, although their magnitude and direction depend on the study system [20,22]. In remnant pest populations, such differences may influence persistence by altering the movement, feeding and reproductive contributions of surviving females and males, but their joint effects remain poorly understood.

Colorado potato beetle (*Leptinotarsa decemlineata* Say, 1824; CPB) is one of the most notorious agricultural pests, having evolved resistance to more than 50 chemical compounds [23]. Following its establishment in Europe in the early 1900s, likely in France, CPB expanded across Eurasia and had reached China by 1993 [24,25]. As a highly invasive, polyphagous defoliator exposed to strong insecticide selection, recurrent bottlenecks and founder events, CPB provides an excellent model for testing how genetic and chemical stressors shape persistence in remnant populations [23]. Although prior selection for imidacloprid tolerance can modify CPB responses to combined insecticide and heat stress [26], it remains unclear how inbreeding and sublethal insecticide exposure influence developmental success, behaviour and reproduction.

Here, we tested how inbreeding and sublethal insecticide exposure shape survival and survivor phenotypes in the CPB, with the aim of understanding how remnant populations may persist after insecticide-driven bottlenecks. We quantified developmental success from larva to adulthood, locomotor activity, leaf consumption and reproductive performance (egg production and hatching success) across four experimental groups: control beetles (never experienced insecticide exposure), control beetles exposed to a sublethal insecticide, inbred beetles generated by two consecutive generations of full-sibling mating and inbred beetles exposed to the sublethal insecticide treatment. Because activity, feeding and reproduction were measured only in adults that survived development and emergence, our design allowed us to distinguish developmental filtering from survivor-based adult responses.

Specifically, we asked the following questions: (i) Do inbreeding and sublethal insecticide exposure reduce developmental success and thereby create a bottleneck before adulthood? (ii) Among surviving adults, do locomotor activity, feeding behaviour and reproductive performance shift in ways consistent with phenotypic plasticity under stress? (iii) Are these survivor-based responses sex-specific, particularly for locomotor activity and feeding?

## 2. Methods

### 2.1. Animal husbandry

CPBs used in these experiments originated from individuals collected in agricultural fields near Ithaca, New York, USA and were later transported to the laboratory in Mainz, Germany. This US-origin strain was imported to establish a genetically diverse [27] founder population for large-scale evolutionary experiments requiring high standing genetic variation. Since its establishment, the strain has become the main laboratory stock used for most experiments in our group, including the present study. Because all experimental groups in this study were derived from the same laboratory population and reared under identical conditions for 12 generations prior to the experiment, any population-of-origin effects are held constant across treatments and cannot explain the inbreeding- or insecticide-driven differences we report. In the laboratory, beetles were maintained in a climate chamber under controlled conditions of 24°C, 60% relative humidity, and a 16 : 8 hours light : dark photoperiod in 1 l plastic boxes each with maximum 20 individuals. They were fed exclusively on organically grown, insecticide-free potato plants of the cultivar Annabelle (Ellenberg's Kartoffelvielfalt GmbH & Co. KG), ensuring that the colony had no history of insecticide exposure under laboratory rearing conditions. To minimize inbreeding, crosses were performed among unrelated individuals from across the available laboratory population whenever possible. All individuals used in the experiments were derived from the field-collected laboratory population and belonged to its 12th lab generation that has never experienced insecticide exposure throughout the 12 lab-reared generations.

### 2.2. Insecticide application

Neonicotinoid insecticide exposure was imposed using technical-grade imidacloprid dissolved in 99% acetone. Neonicotinoids are among the most widely used insecticide classes worldwide, and imidacloprid acts on nicotinic acetylcholine receptors, causing paralysis and ultimately death in insects [28,29]. Although imidacloprid has long been used for the management of CPB, its field efficacy has declined owing to the rapid evolution of resistance in many populations [30]. Moreover, although imidacloprid and two other neonicotinoids are banned or restricted in some regions, including the European Union, its use persists elsewhere [31].

To determine an appropriate sublethal dose, we conducted a topical toxicity assay (following the IRAC susceptibility test method 029 [32]) using 2-day-old larvae derived from the offspring of the single mating pair used to establish each inbred line. Based on these assays, the lowest reliably estimable lethal concentration was the LC20, approximately 2 ppm. Because LC20 values may still impose modest mortality, we used half of this concentration, 1 ppm, as the chemical stress treatment in all experiments (see supplementary material for details).

### 2.3. Experimental design

In all experiments, founders originated from the same laboratory stock population, thereby standardizing pre-experimental rearing conditions across treatment groups. To disentangle the effects of inbreeding and insecticide exposure, we established four treatment groups that differed in lineage history and exposure regime: (i) control beetles with no topical treatment (*inbreeding*−/*insecticide*−), (ii) control beetles exposed to a sublethal dose of imidacloprid during development (*inbreeding*−/*insecticide*+), (iii) inbred beetles produced by two generations of single-pair full-sib mating and treated with acetone alone (*inbreeding*+/*insecticide*−), and (iv) inbred beetles produced by two generations of single-pair full-sib mating and exposed to imidacloprid during development (*inbreeding*+/*insecticide*+; figure 1).

**Figure 1.**
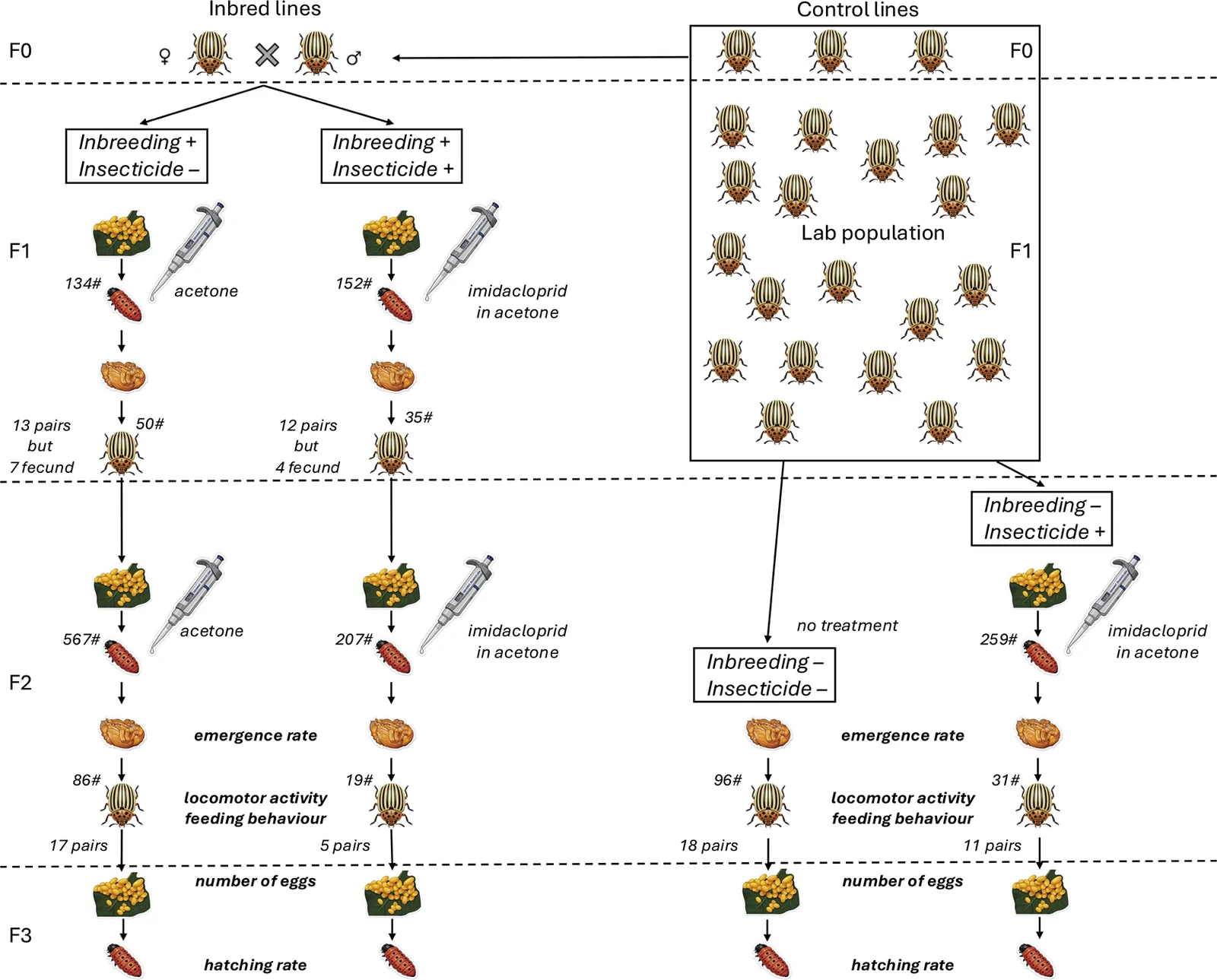
Schematic of the experimental design. Inbred lineages were founded from single mating pairs drawn from the laboratory population and maintained through two generations of full-sib mating. In the *inbreeding*+/*insecticide*− lineage, 2-day-old larvae received topical acetone in both F1 and F2, whereas in the *inbreeding*+/*insecticide*+ lineage, 2-day-old larvae received 1 µl of 1 ppm imidacloprid in acetone in both F1 and F2. In parallel, control beetles from the same laboratory population were used to generate two control-line treatments: an *inbreeding*−/*insecticide*− group that received no topical treatment and an *inbreeding*−/*insecticide*+ group in which 2-day-old F2 larvae received a single topical application of 1 µl of 1 ppm imidacloprid in acetone. The emergence rate was quantified in F2, locomotor activity and feeding behaviour were measured in surviving F2 adults and reproductive output was scored from F3 eggs. Numbers indicate sample sizes at the corresponding developmental stages.

For the inbred treatments, single mating pairs from the laboratory population founded independent lineages in F0. Their F1 offspring were reared separately and, at 2 days after hatching, larvae assigned to the *inbreeding*+/*insecticide*− treatment received a topical application of 1 µl acetone, whereas larvae assigned to the *inbreeding*+/*insecticide*+ treatment received a topical application of 1 µl of 1 ppm imidacloprid dissolved in acetone. Surviving F1 adults were paired with their full siblings to generate the F2 generation, and the same treatment regime was repeated in F2. The F2 inbred generation used in the experiments was established from independently maintained fecund F1 full-sib pairs, yielding seven out of 13 lines in the *inbreeding*+/*insecticide*− treatment and four out of 12 lines in the *inbreeding*+/*insecticide*+ treatment (figure 1).

In parallel, beetles from the same laboratory population were used to establish the two remaining treatment groups. The *inbreeding*−/*insecticide*− group received no topical treatment, whereas 2-day-old larvae in the *inbreeding*−/*insecticide*+ group received a single topical application of 1 µl of 1 ppm imidacloprid dissolved in acetone in F2. Thus, all beetles phenotyped in the main experiment belonged to the F2 generation, but treatment histories differed among groups: the *inbreeding*−/*insecticide*+ group was directly exposed to imidacloprid once in F2, the *inbreeding*+/*insecticide*− group received acetone applications in F1 and F2, and the *inbreeding*+/*insecticide*+ group received imidacloprid in both F1 and F2 (figure 1).

Sample sizes for all traits and treatment groups are provided in table 1. Sample sizes varied among treatment groups, particularly for the inbred, insecticide-exposed lineage, which showed the lowest survival to adulthood (table 1). Because these differences arise directly from the developmental mortality imposed by inbreeding and insecticide exposure, rather than from unequal sampling effort, group sizes could not be equalized without discarding biologically informative variation in survival. We therefore report the realized sample sizes for each group and used statistical approaches (type II tests, negative binomial models and variance-homogeneity checks) suited to unbalanced designs (§2.5). In each generation, 2-day-old larvae were randomly assigned to replicate rearing boxes with 10 larvae per box, except in the *inbreeding*−/*insecticide*+ group, in which 20 larvae were placed in each replicate box to reduce workload. Larval densities used here (10–20 individuals per box) fall within the range routinely used in our laboratory colony, where prior rearing experience across multiple generations indicates no detectable effect of density on survival or reproduction at densities up to 25 individuals per box under *ad libitum* feeding. These densities are also consistent with the natural aggregation of *L. decemlineata* larvae, which hatch in clusters from single egg masses and remain comparatively sedentary relative to adults [33]. Larvae were maintained under standard conditions with potato foliage provided *ad libitum* until the fourth instar. At that stage, larvae were transferred to soil-filled boxes for pupation, with no more than 20 fourth-instar larvae placed in each soil box.

**Table 1.** Sample sizes for each trait measured across the four treatment groups. Values indicate the number of female (F) and male (M) individuals used in locomotor activity and feeding assays, and the number of independent replicates used for reproductive output of surviving F2 pairs and emergence rate. Reproductive output was quantified at the mating-pair level, whereas emergence rate was quantified at the soil-box level.

	number of individuals/replicates			
traits	*inbreeding*− *insecticide*−	*inbreeding*− *insecticide*+	*inbreeding*+ *insecticide*−	*inbreeding*+ *insecticide*+
emergence rate	7	8	14	5
locomotor activity	F: 30 M: 30	F: 11 M: 20	F: 46 M: 40	F: 13 M: 6
feeding behaviour	F: 29 M: 30	F: 11 M: 20	F: 46 M: 40	F: 12 M: 5
reproductive output	18	11	17	5

Lower sample sizes in insecticide-treated groups reflect higher mortality during the final stage of development (pupa to adult) relative to the corresponding controls (supplementary material, table S2).

After adult emergence, the same individuals were assayed sequentially for locomotor activity and feeding behaviour and were subsequently paired within treatment for reproduction. All collected eggs were synchronized for hatching by storage at 10°C for 5 days and sprayed with water upon return to the climate chamber to prevent desiccation. Eggs were stored at 10°C for 5 days to synchronize hatching across lines and treatments, since eggs for each replicate could only be collected from a single mating pair with a limited daily egg output. This storage temperature was applied identically across all treatment groups and follows an established CPB rearing procedure [34]. Individuals were randomized across treatments and assay order throughout the experiment, and blinding was implemented wherever feasible during data collection, analysis and scoring.

Because the *inbreeding*−/*insecticide*− controls were left untreated, whereas the *inbreeding*+/*insecticide*− controls received acetone applications, solvent-handling effects cannot be fully excluded in comparisons involving the untreated controls. However, acetone is highly volatile and has shown no detectable adverse effects in insect topical application assays [35], making substantial solvent effects unlikely. Furthermore, our dedicated acetone-control experiment found no detectable effect of acetone exposure on locomotor activity, feeding or reproductive traits (supplementary material, figures S2–S4).

### 2.4. Quantification of fitness and behavioural traits

We quantified two pre-adult performance measures. First, developmental success was calculated as the percentage of treated 2-day-old larvae that developed into viable adults, using lineage-level larval-to-adult counts (both in the F1 and F2 cohorts; summarized in supplementary material, table S2). Second, the emergence rate was calculated per soil box as the percentage of fourth-instar larvae transferred to soil that successfully emerged as adults (in the F2 cohort). For the *inbreeding*−/*insecticide*− (control group), replicate-level emergence rate data were available for the main analysis, although the starting larval counts required for full larval-to-adult summary reconstruction were not retained for the supplementary count table (supplementary material, table S2).

Freshly emerged F2 adult beetles were then recovered from the soil and sexed under a microscope according to established morphological criteria [36]. Locomotor activity was assessed by placing each beetle individually in a rectangular Petri dish (120 × 120 × 17 mm with vents) with a 1 × 1 cm grid on the base. Individuals were allowed to acclimate for 1 minute before the assay began, after which the number of grid lines crossed during the subsequent 3 minutes was recorded. This acclimation period was included to distinguish general locomotor activity from initial exploratory behaviour [37]. The total number of line crossings was used as the activity score, with higher values indicating greater locomotor activity.

Immediately afterwards, feeding behaviour was quantified in the same surviving F2 adults by placing each beetle individually on a standardized potato leaf disc (diameter = 25.0 mm; area approx. 490.87 mm²) for 1 hour. Leaf discs were photographed before and after beetle exposure to calculate the area consumed (see §2.5 below). After the feeding assay, beetles were paired as single female–male replicates within treatment and maintained with food provided *ad libitum* to allow mating and oviposition.

Reproductive performance was then quantified from eggs produced by these surviving F2 adults. Once oviposition began, F3 eggs were collected over a period of 8 days, and the total number of eggs laid per female during this period was recorded. To synchronize egg hatching time, collected eggs were kept at 10°C for 5 days and sprayed with water to prevent desiccation before being returned to the climate chamber. Eggs were monitored for four weeks, and hatched larvae were counted and removed to minimize cannibalism. The hatching success of F3 eggs was calculated as the number of hatched larvae divided by the total number of eggs laid by each pair. Because emergence and developmental success were reduced by inbreeding and insecticide exposure, the F2 adults available for behavioural and reproductive assays represent only the subset that survived this developmental filter. Trait values reported for locomotor activity, feeding, egg production and hatching success should therefore be interpreted as survivor-based phenotypes, not as estimates of reproductive output across the full initial F2 cohort; higher performance among survivors in a given treatment does not imply a net reproductive benefit of that treatment at the population level, since fewer individuals from that treatment reached reproductive age at all.

### 2.5. Data acquisition, image and statistical analysis

All statistical analyses were conducted in R version 4.5.2 [38] through RStudio version 2026.01.0+392 [39], and plots were generated using the *ggplot2* [40] and *viridis* [41] packages. Experimental groups were analysed as a factorial 2 × 2 design, with inbreeding status (control versus inbred), insecticide exposure (no versus yes) and their interaction fitted as fixed effects, while sex was included where applicable. Predictor significance was assessed using type II tests implemented in the *car* package [42].

Developmental success of the F2 cohort was summarized descriptively from larval-to-adult counts across treatments and generations (supplementary material, table S2). The emergence rate was analysed with a linear model from the *stats* package [38], including inbreeding, insecticide exposure and their interaction, after checking variance homogeneity across groups with Levene's tests and inspection of group-specific variances [42].

Locomotor activity of F2 adults was analysed using negative binomial generalized linear models from the *MASS* package [43]. The full model included inbreeding, insecticide exposure, their interaction, sex and consumed leaf area as predictors, and additional sex-specific models were fitted separately for females and males using inbreeding, insecticide exposure and their interaction.

Leaf area consumption by F2 beetles was quantified using a custom automated pipeline (see supplementary material for more details) developed in the R statistical environment [38]. Image processing was performed using the *EBImage* [44] and *stringr* [45] packages in R to analyse paired photographs taken before and 1 hour after the feeding trial. To ensure robust segmentation of leaf material, an iterative thresholding approach was applied. Images were converted to greyscale, and binary masks were generated using a sequence of intensity thresholds (0.60, 0.65, 0.70 and 0.75). Morphological operations, including opening and hull-filling, were applied to remove noise and close internal gaps within the leaf tissue. The median pixel area derived from these iterations was selected as the final measurement to minimize detection artefacts. Calibration was performed individually for each experimental replicate to control for minor variations in camera distance. A pixel-to-mm² conversion factor was derived from the ‘before’ image of each pair based on the known surface area of the standard leaf discs (diameter = 25.0 mm; Area ≈ 490.87 mm^2^). This conversion factor was subsequently applied to the corresponding ‘after’ image.

Leaf consumption *C* (in mm^2^) was calculated as:

$$\begin{array}{r} C=A_{initial}-A_{after}=A_{initial}\left(1-\frac{P_{after}}{P_{initial}}\right) \end{array}$$

where *A*_initial_ is the fixed area of the leaf disc (490.87 mm²) and *P*_initial_ and *P*_after_ are the leaf-pixel counts before and after feeding, respectively. Consumption was also expressed as a percentage of the total available leaf area. The pipeline automatically matched image pairs based on encoded file identifiers for all four treatment groups. For feeding behaviour, both consumed leaf area (mm²) and percentage leaf area consumed were analysed using linear models from the *stats* package in R [38]. Variance heterogeneity was assessed with Levene's tests and by inspection of group-specific variances (package *car*; [42]), and feeding variables were therefore analysed on the log scale. Consumed leaf area (mm²) was modelled as a function of inbreeding status, insecticide exposure, their interaction, sex and locomotor activity, whereas percentage leaf area consumed was modelled as a function of inbreeding status, insecticide exposure, their interaction and sex.

Reproductive traits were analysed at the pair level. The hatching success of F3 eggs produced by surviving F2 pairs was analysed with a linear model from the *stats* package [38] including inbreeding status, insecticide exposure, their interaction, female activity, male activity, female feeding and male feeding as predictors. Total egg production by surviving F2 pairs was analysed using a negative binomial generalized linear model from the *MASS* package [43] with the same predictor set.

For the topical toxicity assay to calculate the LC20 values, mortality data were analysed with a binomial generalized linear model with a probit link (*stats* package; [38]), using the numbers of dead and alive larvae at each concentration, and lethal concentrations were obtained from that fitted model.

## 3. Results

Inbreeding and sublethal insecticide exposure first acted as a strong developmental filter, reducing emergence before adulthood. The emergence rate was highest in *inbreeding*−/*insecticide*− controls and was reduced by both inbreeding and insecticide exposure, with no significant interaction between the two stressors. Among the F2 adults that survived this bottleneck, responses were trait-specific: male locomotor activity was reduced by both stressors, female and male feeding increased under both, hatching success was reduced by inbreeding but higher under insecticide exposure, and total egg production was reduced by inbreeding only. Across all analysed traits, no significant inbreeding-by-insecticide interaction was detected. Because activity, feeding and reproduction were measured only after emergence, these adult responses are best interpreted as survivor-based phenotypes and may be consistent with phenotypic plasticity under environmental stress.

### 3.1. Developmental success

Developmental success from F2 larvae to viable F2 adults declined under both inbreeding and insecticide exposure (supplementary material, table S2). Across the inbred lineages, the proportion of larvae reaching viable adulthood declined from 37.3 to 23% in F1 and from 15.2 to 9.2% in F2, while 12% of larvae reached adulthood in the F2 insecticide-exposed treatment (*inbreeding*−/*insecticide*+). The emergence rate was highest in control (*inbreeding*−/*insecticide*−) and lower in all other groups, with significant main effects of inbreeding and insecticide exposure but no significant interaction in the factorial model (figure 2; table 2). These results indicate that both genetic stress and chemical stress reduced emergence, but their effects were primarily additive. Because adult behavioural and reproductive traits were measured only after this developmental bottleneck, downstream adult responses reflect survivor-based phenotypes after developmental filtering.

**Figure 2.**
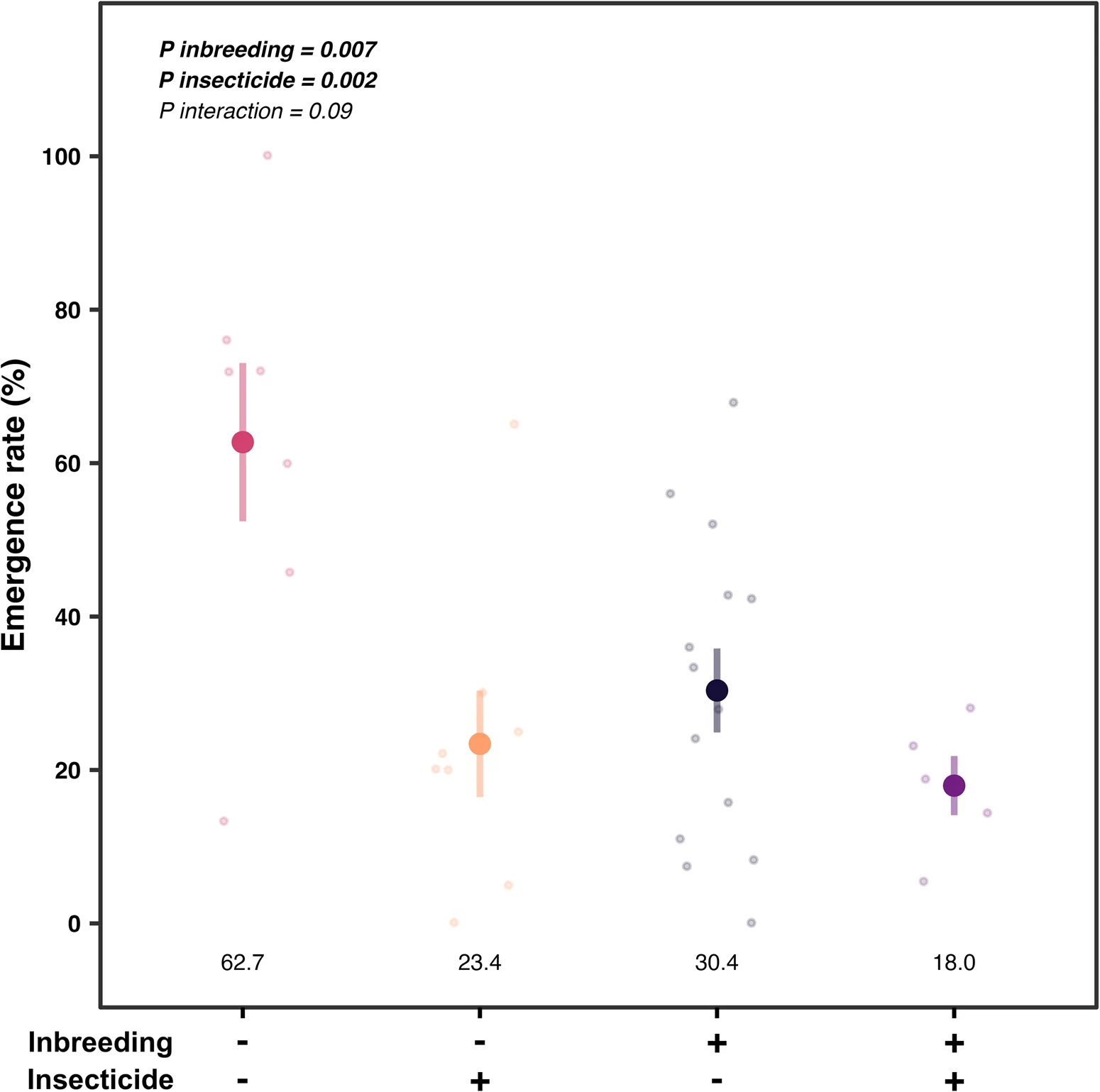
Emergence rate (%) across the four combinations of inbreeding status and insecticide exposure: (i) *inbreeding*−/*insecticide*−, (ii) *inbreeding*−/*insecticide*+, (iii) *inbreeding*+/*insecticide*−, and (iv) *inbreeding*+/*insecticide*+. Points show replicate soil boxes, and larger points with error bars indicate means ± SE; numbers above the x-axis give the corresponding group means. In-panel *P*-values are from the factorial model, including inbreeding, insecticide exposure and their interaction.

**Table 2.** Summary of factorial model results testing the effects of inbreeding, insecticide exposure and their interaction on developmental, behavioural and reproductive traits. Locomotor activity was analysed separately for females and males because sex was significant in the full model. Statistics are presented as *F*-values for linear models and likelihood-ratio $\chi^{2}$ values for negative binomial models, with associated *p*-values.

Traits	*inbreeding*	*insecticide*	*inbreeding* × *insecticide*
emergence rate	***F* _1,30_ = 8.5, *p* = 0.007**	***F* _1,30_ = 11.6, *p* = 0.002**	*F* _1,30_ = 3.1, *p* = 0.09
locomotor activity	females: *χ*_1_^2^ = 0.2, *p* = 0.67; **males: *χ*_1_^2^ = 4.1, *p* = 0.04**	females: *χ*_1_^2^ = 0.9, *p* = 0.34; **males: *χ*_1_^2^ = 7.7, *p* = 0.006**	females: *χ*_1_^2^ = 1.2, *p* = 0.28; males: *χ*_1_^2^ = 0.1, *p* = 0.78
feeding behaviour	***F* _1,123_ = 21.4, *p* < 0.0001**	***F* _1,123_ = 9.5, *p* = 0.003**	*F* _1,123_ = 0.2, *p* = 0.64
hatching rate	*F* _1,42_ = 7.0^*^, ***p* = 0.01**	***F* _1,42_ = 6.8, *p* = 0.01**	*F* _1,42_ = 0.02, *p* = 0.88
total number of eggs laid	***χ*_1_^2^ = 11.4, *p =* 0.0007**	*χ*_1_^2^ = 0.2, *p* = 0.67	*χ*_1_^2^ = 0.9, *p* = 0.35

^*^ One reproductive pair (control, *inbreeding*−/*insecticide*−) was excluded from the hatching-rate model owing to a missing female feeding measurement, yielding *n* = 50 pairs (d.f. = 42) for this analysis. All other models used the full sample of 51 pairs. Excluding this pair did not change the direction or significance of any effect; a sensitivity check including this pair (with the missing predictor removed) yielded qualitatively identical results.

### 3.2. Locomotor activity

Locomotor activity of surviving F2 beetles differed strongly between the sexes, with females more active than males overall (figure 3). In the full model, sex (*χ*_1_^2^ = 50.3, *p* < 0.0001) and insecticide exposure (*χ*_1_^2^ = 6.3, *p* = 0.01) significantly affected activity, whereas inbreeding (*χ*_1_^2^ = 1.3, *p* = 0.24) and the inbreeding-by-insecticide interaction (*χ*_1_^2^ = 1.8, *p* = 0.18) did not. Because sex had a significant effect in the full model, locomotor activity was subsequently analysed separately for females and males. Sex-specific analyses showed no significant effects of inbreeding, insecticide exposure or their interaction in females (figure 3; table 2). In males, however, both inbreeding and insecticide exposure significantly reduced activity, with no evidence of an interaction between them (figure 3; table 2). Mean male activity declined from 11.3 in control (*inbreeding*−/*insecticide*−) to 3.5 in insecticide-exposed beetles (*inbreeding*−/*insecticide*+), 5.7 in inbred beetles (*inbreeding*+/*insecticide*−) and 1.3 in inbred insecticide-exposed beetles (*inbreeding*+/*insecticide*+).

**Figure 3.**
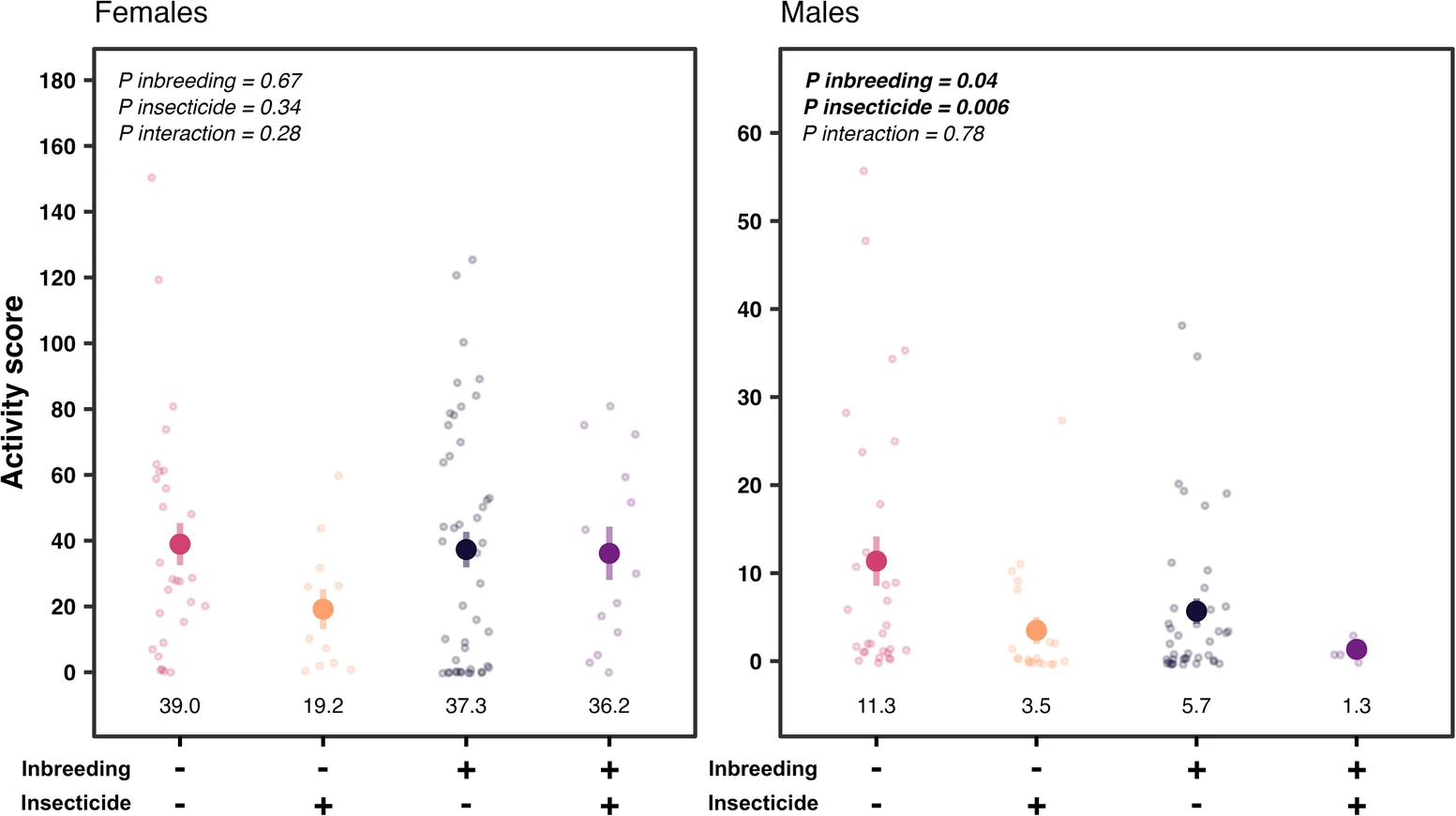
Locomotor activity (activity score) of surviving F2 females (left) and males (right) across the four combinations of: (i) *inbreeding*−/*insecticide*−, (ii) *inbreeding*−/*insecticide*+, (iii) *inbreeding*+/*insecticide*−, and (iv) *inbreeding*+/*insecticide*+. Points show individual beetles, and larger points with error bars indicate means ± SE; numbers above the *x*-axis indicate group means. *P*-values shown within each panel are from the sex-specific factorial models testing inbreeding, insecticide exposure and their interaction.

### 3.3. Feeding behaviour

Leaf consumption by surviving F2 beetles increased with both inbreeding and insecticide exposure (figure 4). In the factorial model for consumed leaf area, both inbreeding and insecticide exposure had significant effects, whereas their interaction and the effect of sex were not significant (figure 4; table 2). Mean consumed leaf area increased from 96.9 mm² in controls (*inbreeding*−/*insecticide*−) to 119.2 mm² in insecticide-exposed beetles (*inbreeding*−/*insecticide*+), 146.5 mm² in inbred beetles (*inbreeding*+/*insecticide*−) and 194.9 mm² in inbred insecticide-exposed (*inbreeding*+/*insecticide*+) beetles. The same pattern was evident when consumption was expressed as percentage leaf area consumed, increasing from 19.7% in controls (*inbreeding*−/*insecticide*−) to 24.3, 29.9 and 39.7% across the remaining treatment combinations. Thus, both genetic stress and chemical stress were associated with increased feeding among surviving adults, but there was no evidence that their combined effect differed from additivity. Because feeding was measured only after the developmental bottleneck, these estimates reflect survivor-based adult feeding behaviour rather than full-cohort herbivory.

**Figure 4.**
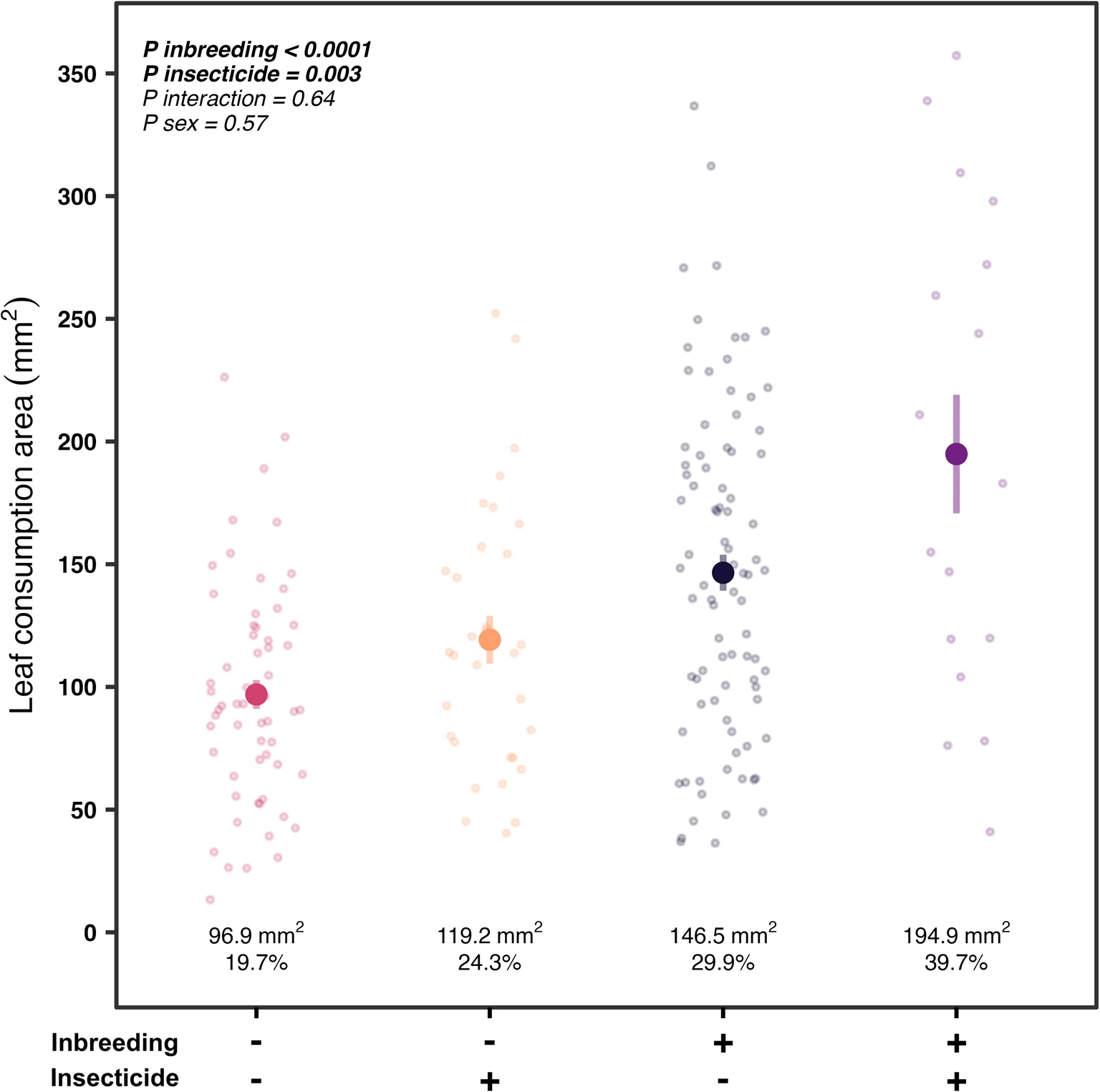
Leaf consumption area (mm²) of surviving F2 beetles across the four combinations of inbreeding status and insecticide exposure: (i) *inbreeding*−/*insecticide*−, (ii) *inbreeding*−/*insecticide*+, (iii) *inbreeding*+/*insecticide*−, and (iv) *inbreeding*+/*insecticide*+. Points show individual beetles, and larger points with error bars indicate means ± SE. Numbers above the *x*-axis indicate group means for consumed leaf area in mm² and, on the second row, the corresponding percentage of leaf area consumed. *P*-values shown within the panel are from the factorial model testing inbreeding, insecticide exposure, their interaction and sex.

### 3.4. Reproductive output

Among surviving F2 pairs, the hatching success of F3 eggs was affected by both inbreeding and insecticide exposure, whereas their interaction was not significant (figure 5; table 2). Mean hatching success was 43.7% in controls (*inbreeding*−/*insecticide*−), 70.4% in insecticide-exposed pairs (*inbreeding*−/*insecticide*+), 22.7% in inbred pairs (*inbreeding*+/*insecticide*−) and 50.4% in inbred insecticide-exposed pairs (*inbreeding*+/*insecticide*+). Total egg production showed a different pattern: egg number was significantly reduced by inbreeding, whereas insecticide exposure and the inbreeding-by-insecticide interaction were not significant in the fitted model (figure 5; table 2). Descriptively, mean egg production was highest in insecticide-exposed pairs (*inbreeding*−/*insecticide*+; 257.0 eggs), followed by controls (*inbreeding*−/*insecticide*−; 211.4 eggs), inbred insecticide-exposed pairs (*inbreeding*+/*insecticide*+; 139.8 eggs) and inbred pairs (*inbreeding*+/*insecticide*−; 96.4 eggs). Because these reproductive traits were measured only in adults that survived development and emergence, they should be interpreted as survivor-based reproductive performance rather than as unconditional benefits of insecticide exposure across the full cohort.

**Figure 5.**
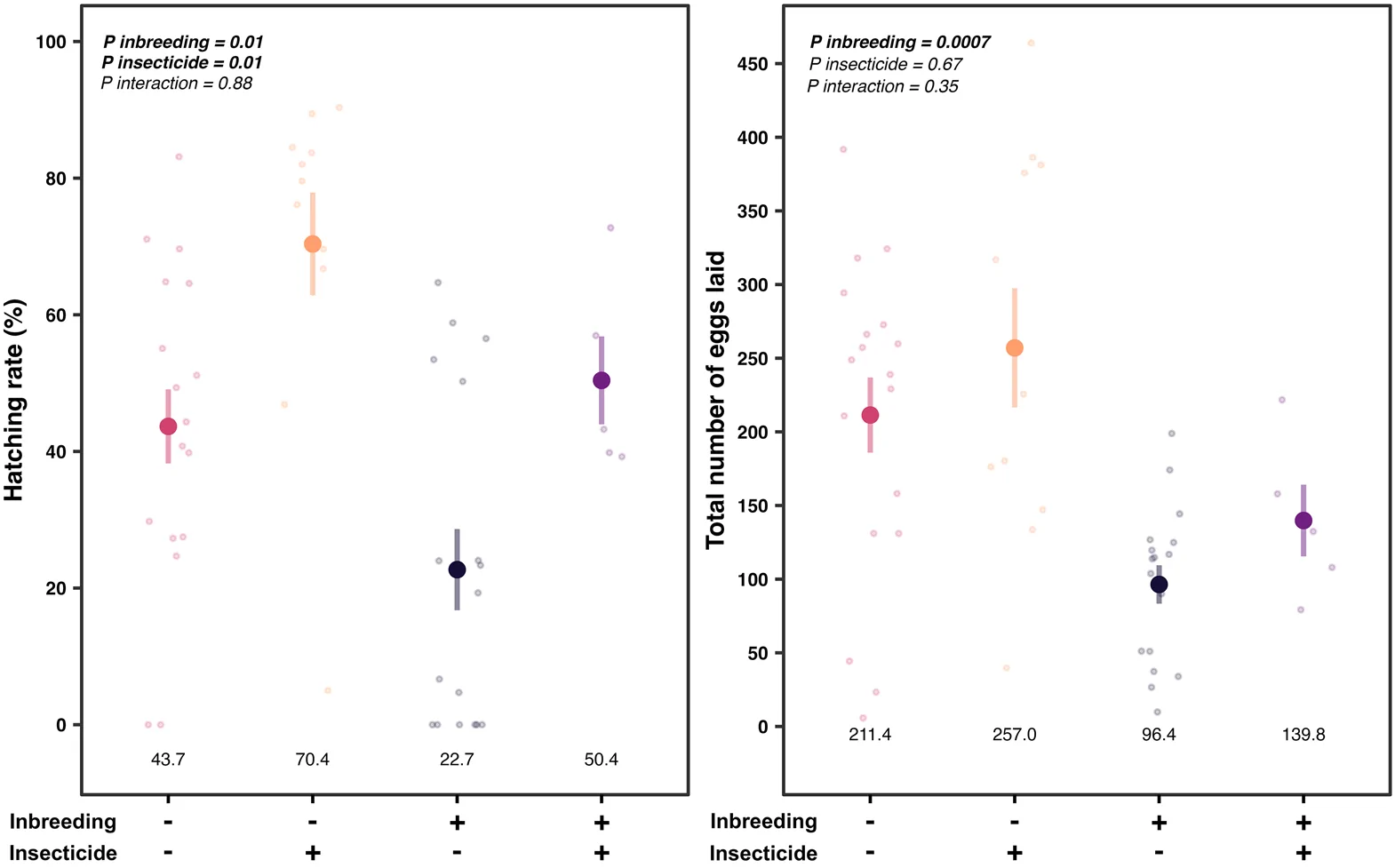
Reproductive output of surviving F2 pairs across the four combinations of inbreeding status and insecticide exposure: (i) *inbreeding*−/*insecticide*−, (ii) *inbreeding*−/*insecticide*+, (iii) *inbreeding*+/*insecticide*−, and (iv) *inbreeding*+/*insecticide*+. The left panel shows the hatching success (%) of F3 eggs, and the right panel shows the total number of eggs laid. Points show individual mating pairs, and larger points with error bars indicate means ± SE; numbers above the *x*-axis indicate group means. The panels are arranged by the factorial combination of inbreeding status and insecticide exposure.

### 3.5. Correlations among measured traits

To assess associations between behavioural and reproductive traits, we fitted models for hatching success and total egg production that included sex-specific parental locomotor activity and feeding as predictors. We found no evidence that parental activity or feeding significantly predicted either reproductive trait (*p* = 0.4–0.9). Thus, within the fitted models, variation in hatching success and egg production was not explained by the measured behavioural traits of surviving parents.

## 4. Discussion

In our experiments, inbreeding and sublethal insecticide exposure created a strong developmental bottleneck, but the adults that survived retained substantial capacity to feed and reproduce. Persistence after insecticide-driven bottlenecks therefore appears most likely under two conditions: when beetles avoid the fitness costs of inbreeding, and when survivors can adjust their behaviour and physiology in response to continued stress. We refer to this second capacity as phenotypic plasticity, evidenced in our data by survivors showing increased feeding and, in some cases, increased reproductive performance under stress rather than a uniform decline in function. From an agricultural perspective, this means that short-term suppression may be partly offset by remnant beetles that survive treatment and continue to contribute to herbivory and population recovery.

The clearest consequence of stress in our study was the strong developmental filtering that occurred before adulthood. Inbreeding and sublethal insecticide exposure both reduced developmental success, and inbreeding also depressed later-life reproductive traits, particularly hatching success and egg production, indicating that remnant populations are more likely to persist when beetles avoid the costs of mating among relatives. This pattern is broadly consistent with studies showing that environmental challenge can magnify the fitness consequences of inbreeding, commonly used as a proxy for reduced genetic quality: elevated temperature reduced fitness more strongly in inbred than in outbred seed beetles, with detrimental effects on eclosion probability and lifetime reproductive success becoming more pronounced under thermal stress [15], and nutritional stress increased inbreeding depression in *Drosophila melanogaster*, particularly for egg-to-adult viability, with stronger effects at higher stress levels [16]. Although these studies involved thermal and nutritional stress rather than insecticide exposure, they support the broader view that environmental challenge can exacerbate the effects of inbreeding and help explain why bottlenecked beetle populations may suffer substantial losses before adulthood. At the same time, such effects are not universal, as reviews and meta-analyses show that the magnitude and even direction of inbreeding-by-stress interactions vary across stress types, traits, life stages and study systems [46–48]. Taken together, our results suggest that outbreeding is likely to be especially important for persistence after insecticide-driven bottlenecks because it reduces the fitness costs imposed by low genetic quality during development and reproduction.

The second major pattern in our study was that the beetles surviving the developmental bottleneck showed adult responses consistent with phenotypic plasticity under stress [49,50]. Among these survivors, feeding increased under both inbreeding and insecticide exposure and hatching success was higher in insecticide-exposed pairs, whereas egg production appears to be higher under insecticide exposure but did not show a significant insecticide effect in the fitted model. One possible explanation is that surviving adults adjusted resource allocation after stress exposure, either through hormetic responses (i.e. a beneficial effect at low doses but toxicity at higher doses [51]) or through stress-induced allocation shifts [52], although our data do not allow us to distinguish between these mechanisms. This interpretation is consistent with broader evidence that sublethal pesticide exposure can modify insect behaviour and resource-acquisition traits, including feeding-related responses [22]. It also differs from previous work in CPB under insecticide tolerance-by-heat stress, where herbivory was generally reduced and fecundity remained largely unchanged, and fits the broader idea that feeding responses can depend strongly on when herbivory is measured relative to exposure, with acute suppression during exposure but delayed compensatory feeding in survivors [26,53,54]. In that sense, our results suggest that remnant beetles may persist not only because some individuals survive the initial bottleneck, but also because those survivors can adjust feeding and retain substantial reproductive capacity under continued stress.

Activity responses were strongly sex-specific in our study, with female locomotor activity remaining comparatively stable whereas male activity declined under both inbreeding and insecticide exposure. This pattern suggests that the behavioural costs of stress were concentrated in males, indicating a sex- and trait-specific response rather than a general reduction in activity across all surviving adults. In the context of a bottlenecked remnant population, reduced male locomotor activity could be important because it may lower dispersal and favour local reproduction under continued sublethal exposure, although this demographic consequence remains to be tested directly. Our results are consistent with broader evidence that sublethal insecticide exposure can disrupt insect behaviour, including locomotion and activity [55–57], but they also support the view that sex differences in stress sensitivity are context-dependent rather than following a uniform pattern [20,58–60]. Thus, in CPBs, reduced male activity may represent another way in which survivor phenotypes contribute to persistence after developmental filtering.

From an agricultural perspective, our results suggest that insecticide application may achieve short-term demographic suppression without delivering equally strong longer-term control. Although fewer beetles completed development under insecticide exposure, the remnant adults that survived fed more and retained substantial reproductive capacity, including higher hatching success among insecticide-exposed pairs. This means that a numerically reduced survivor population may still impose considerable herbivory and contribute to renewed population growth, potentially weakening the overall benefits of insecticide application [61–63]. In this sense, the persistence of remnant populations may depend not only on the immediate lethality of insecticide treatment, but also on whether surviving beetles are outbred and able to respond plastically under continued stress.

Our design contrasted two discrete points along what is likely a continuum of relatedness in natural populations: full-sib mating over two generations versus mating among a comparatively large and less related laboratory stock. In the field, the degree of inbreeding experienced by a remnant population after an insecticide-driven bottleneck will depend on how many breeding individuals survive and how spatially or temporally isolated they remain; smaller, more isolated remnants are expected to accumulate higher relatedness over consecutive generations than larger or better-connected ones. We are not aware of an established relatedness threshold separating ‘inbred’ from ‘outbred’ CPB populations in the field; rather, based on general patterns in the inbreeding-depression literature, fitness costs are expected to scale continuously with the degree of relatedness rather than switching on above a fixed cut-off [9,46]. Our results, obtained at the more extreme end of this continuum, should therefore be interpreted as demonstrating that inbreeding can impose substantial costs when it occurs, rather than establishing the precise relatedness level at which such costs first become detectable in natural populations. Testing intermediate degrees of relatedness will be an important next step in extending these findings to field-realistic bottleneck scenarios.

Several features of our design warrant caution in interpretation. First, exposure history differed among groups: the *inbreeding*−/*insecticide*+ treatment involved a single direct exposure in F2, whereas the *inbreeding*+/*insecticide*+ lineages experienced imidacloprid in both F1 and F2, and the *inbreeding*+/*insecticide*− lineages received acetone applications in both generations while the *inbreeding*−/*insecticide*− controls remained untreated. Second, the experiment was conducted in a single laboratory population and at a single sublethal imidacloprid dose, which limits direct inference about other populations, resistance backgrounds and exposure regimes. These features mean that the study is best interpreted as testing how lineage history and sublethal exposure shape developmental filtering and survivor phenotypes under this specific experimental framework, rather than as a fully symmetric solvent-controlled test of all possible treatment histories. Nevertheless, the central message remains robust because the main effects of inbreeding and insecticide exposure were consistent across multiple endpoints, including reduced emergence, increased feeding, reduced male locomotor activity and reduced reproductive performance in inbred beetles, while no significant inbreeding-by-insecticide interaction was detected across the analysed traits.

Despite these design-specific limitations, our study identifies a general process by which stress can reshape pest populations through developmental filtering and survivor-based responses. In CPBs, persistence after insecticide-driven bottlenecks appears to depend on two features in particular: avoiding the fitness costs of inbreeding and retaining the capacity for phenotypic plasticity among survivors. This is consistent with the observed high genetic diversity and phenotypic plasticity in this pest [27,64]. More broadly, our results suggest that the consequences of insecticide exposure may depend not only on how many individuals are removed, but also on which individuals survive and what they are able to do afterwards. Future work across multiple populations, resistance backgrounds and environmental contexts will be needed to determine how general these bottleneck-driven dynamics are and whether similar processes shape persistence in other stressed populations. Although our experiment was not designed to identify mechanism, one possibility is that inbreeding and sublethal insecticide exposure affect overlapping physiological pathways involved in neural signalling, detoxification or general stress tolerance rather than acting through a single shared target site [9,65,66]. Testing this idea will require genomic or transcriptomic comparisons of lineages that differ in breeding status and insecticide exposure history, particularly for nicotinic acetylcholine receptor subunits and detoxification- or stress-response genes [65–67]. Understanding persistence after control therefore requires attention not only to mortality, but also to the genetic quality and plasticity of the remnant population.

## 5. Conclusion

Our study shows that inbreeding and sublethal insecticide exposure do not affect CPB performance uniformly but instead create a sequence of developmental filtering followed by survivor-based adult responses. Developmental success and emergence were reduced, whereas among the adults that survived this bottleneck, male locomotor activity declined, feeding increased and substantial reproductive capacity was retained. Together, these findings suggest that persistence after insecticide-driven bottlenecks is most likely when beetles avoid the fitness costs of inbreeding and when survivors can respond plastically to stress. Persistence after control depends not only on how many beetles are removed, but also on the genetic quality and plasticity of those that remain.

## Supplementary Material



## Data Availability

Data and code generated and analysed during this study are available in the supplementary section [68].
