# Supplementary material for: Sublethal insecticide exposure and inbreeding impose a developmental bottleneck but enhance crop-damaging traits in surviving Colorado potato beetles
Source: R Soc Open Sci. 2026 Nov 4;13(11):261014. doi: 10.1098/rsos.261014 (PMC13730666; doi:10.1098/rsos.261014)
Supplement: Supplementary file 1 [file rsos.261014.zip › rsos.261014/rsos261014_si_001.pdf]

SUPPLEMENTARY INFORMATION

Sublethal insecticide exposure and inbreeding impose a developmental bottleneck but enhance crop-damaging traits in surviving Colorado potato beetles

Nijat Nariman*, Himrekha Agarwal and Shuqing Xu

Address: Institute of Organismic and Molecular Evolution (iomE), Johannes Gutenberg-University of Mainz, 55128 Mainz, Germany.

* Correspondence and present address: Nijat Nariman; Institute of Organismic and Molecular Evolution (iomE), Johannes Gutenberg-University of Mainz, Hanns-Dieter-Hüsch-Weg 15, Mainz, 55128, Germany. Email: nijat.nariman@gmail.com / nnariman@uni-mainz.de

Journal: Royal Society Open Science

Article DOI: 10.1098/rsos.261014

1. Topical toxicity assay following IRAC Method 029

1.1. Larval origin and rearing

The topical toxicity assay used to establish the sublethal insecticide dose was conducted on Colorado potato beetle, *Leptinotarsa decemlineata*, larvae derived from the same laboratory population as the individuals used in the main experiment. For each inbred line, eggs were collected from the single mating pair used to found the line, and the resulting offspring were reared under the standard conditions described in the main text (24°C, 60% relative humidity, 16 h : 8 h light : dark photoperiod) on organically grown, insecticide-free potato plants (cv. Annabelle; Ellenbergs Kartoffelvielfalt GmbH & Co. KG). Larvae were maintained with foliage *ad libitum* until 2-day-old and were then immediately used in the assay.

1.2. Insecticide and test solutions

Technical-grade imidacloprid was dissolved in 99% acetone to prepare the test solutions. A stock solution was first prepared and then serially diluted with acetone to generate the required concentration series. Acetone was used as the solvent because technical-grade imidacloprid dissolved readily in it, allowing rapid

preparation of a homogeneous concentration series (1,2). In addition, acetone is highly volatile and, in insect topical application assays, has been reported to cause no detectable negative effects on key fitness and behavioural traits, making substantial solvent-related effects unlikely (3). Specifically, Jatsch and Ruther (3) found that topical acetone application in *Nasonia giraulti* had no negative effects on longevity, offspring number, mating behaviour, or male pheromone titres. Moreover, our additional experiment investigating effects of acetone on beetles' behavioural and fitness traits provided no evidence that acetone exposure alone altered adult locomotor activity, feeding behaviour, egg production, or hatching success (Fig. S2-S4). Prior to the main assay, preliminary range-finding tests were carried out to determine the lowest concentration inducing complete mortality; concentrations of 100, 50, 25, 20 and 15 ppm were tested. These preliminary tests identified 20 ppm as the minimum concentration causing 100% mortality. The concentrations used in the main assay were then selected following IRAC Susceptibility Test Method 029, which recommends a range of at least 5–6 doses spanning mortality from 0 to 100%. The final nominal concentrations tested were 0, 0.5, 1, 2, 3, 5, 7, 10, 13, 17 and 20 ppm. Acetone alone (0 ppm) served as the solvent control, and all solutions were prepared fresh on the day of use.

1.3. Experimental design and topical application procedure

We followed the IRAC 029 protocol for topical application. For each concentration, four independent replicates were set up, each consisting of five larvae (total N = 20 larvae per concentration). Larvae were randomly assigned to concentrations to avoid bias among doses. Larvae were placed in clean 9 cm Petri dishes lined with filter paper and provided with a fresh potato leaf. Each dish contained five larvae. A standard laboratory micropipette was used to apply 1 µl of the test solution directly to the dorsal side of each larva. This dose volume is within the IRAC recommended range for small larvae and ensures rapid absorption without visible runoff. Control larvae were treated identically with 1 µl of pure acetone. Petri dishes were maintained under the same controlled environmental conditions as the stock colony. Mortality and sublethal effects were scored 48 h after treatment.

S1.4. Mortality scoring and raw data structure

For each replicate at each concentration, we recorded the total number of larvae together with the numbers classified as alive, dead, or severely intoxicated but still capable of only very limited movement. Following IRAC scoring criteria, dead larvae and severely intoxicated larvae were both treated as functionally dead for the dose–response analysis. Percentage mortality was calculated as the number of functionally dead larvae divided by the total number of larvae in the replicate and multiplied by 100, where the total number of larvae per replicate was five. Mortality values spanned the full response range, from 0% at the lowest concentrations to 100% at the highest concentrations.

1.5. Statistical analysis

All statistical analyses were performed in R version 4.5.2 using base functions from the *stats* package (4). The relationship between imidacloprid concentration and larval mortality was analysed using a binomial generalized linear model with a probit link (4), in which the numbers of dead and surviving larvae at each concentration were treated as binomial response data conditional on the total number tested [cbind(dead, alive) ~ dose]. From the fitted model, we derived the lethal concentrations associated with 20%, 30%, 50%, 70%, 95% and 99% mortality (LC20, LC30, LC50, LC70, LC95 and LC99).

1.6. Selection of the sublethal dose for the main experiment

The probit dose–response model estimated an LC20 of 2.2 ppm for 2-day-old larvae (s.e. = 0.7 ppm), with the corresponding lethal concentrations rising to 3.9, 6.8, 9.6, 15.8 and 19.5 ppm for LC30, LC50, LC70, LC95 and LC99, respectively (Fig. S1; Table S1). To impose a mild but repeatable chemical challenge in the main experiment without inducing substantial mortality, we selected a sublethal dose equal to half of the estimated LC20. This concentration was rounded to 1 ppm imidacloprid and applied topically to 2-day-old larvae assigned to the *inbreeding – / insecticide* + and *inbreeding + / insecticide* + treatments.

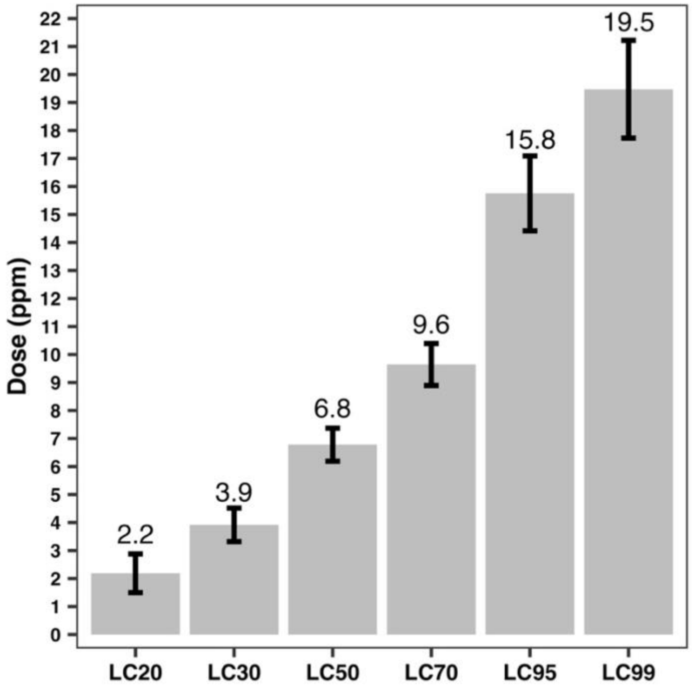

Figure S1. Estimated imidacloprid concentrations (ppm) required to achieve defined mortality thresholds in 2-day-old *Leptinotarsa decemlineata* larvae. Bars represent the estimated lethal concentrations for LC20, LC30, LC50, LC70, LC95 and LC99 derived from a binomial generalized linear model fitted with a probit link. Error bars show the standard errors of the concentration estimates. Numeric labels above the bars indicate the estimated concentrations in ppm, rounded to one decimal place.

Table S1. Estimated imidacloprid concentrations (ppm) required to produce defined mortality levels (LC20, LC30, LC50, LC70, LC95 and LC99) in 2-day-old *Leptinotarsa decemlineata* larvae, together with the corresponding standard errors from the fitted probit model.

Mortality	Dose	Standard error
LC20	2.2	0.7
LC30	3.9	0.6
LC50	6.8	0.6
LC70	9.6	0.8
LC95	15.8	1.3
LC99	19.5	1.7

Table S2. Numbers of larvae (L), females (F), males (M), total adults (T), and developmental success (%) in the F1 and F2 generations across treatments. The data for *inbreeding – / insecticide* – controls and F1 *inbreeding – / insecticide* + treatment were not available.

Treatment	F1					F2				
	L	F	M	T	%	L	F	M	T	%
Inbreeding + *Insecticide –*	134	31	19	50	**37.3**	567	46	40	86	**15.2**
Inbreeding + *Insecticide +*	152	22	13	35	**23**	207	13	6	19	**9.2**
Inbreeding – *Insecticide +*	–	–	–	–	–	259	11	20	31	**12**

For the F2 inbreeding – / insecticide – treatment, the starting number of larvae assigned to soil boxes was not retained in the summary records used for this supplementary table, so larval-to-adult count summaries could not be reconstructed here. However, replicate-level emergence success data for the F2 controls (inbreeding – / insecticide –) were available and were used in the main-text emergence analysis (Fig. 2).

2. Acetone-control experiment

To assess whether acetone exposure during development itself affected adult behavioural or reproductive traits, we conducted an additional solvent-control experiment comparing acetone-treated beetles with untreated controls.

2.1. Methods

Larvae for this experiment were derived from the same laboratory population as the main experiment, but from its seventeenth lab generation (rather than the twelfth generation used in the main experiment), reared under identical standard conditions (24°C, 60% relative humidity, 16h:8h light:dark photoperiod, *ad libitum* potato foliage; 20 larvae per box). A total of 60 two-day-old larvae were topically treated with 1 µl acetone, of which 43 reached the fourth instar and were transferred to soil for pupation, and 34 successfully emerged as adults. In parallel, 60 two-day-old larvae were assigned to the untreated control group without any topical treatment, of which 37 reached the fourth instar and were transferred to soil, and 29 emerged as adults.

Adult beetles were assessed for locomotor activity, feeding behaviour, and reproductive performance using the same general procedures as in the main experiment. Locomotor activity was quantified as the number of grid lines crossed during a 3-min observation period following a 1-min acclimation period. Activity was measured in 19 control beetles (9 females and 10 males) and 20 acetone-treated beetles (10 females and 10 males).

Immediately after the activity assay, beetles were individually provided with a standardised potato leaf disc for 1 h. The initial disc area was standardised as in the main experiments (diameter = 25.0 mm; area approx. 490.87 mm²) and remaining leaf area was quantified using the same general procedures as in the main experiment.

For reproductive measurements, females were paired with males from the same treatment group and maintained by feeding *ad libitum*. The eggs were recorded on three consecutive days. Total egg production was calculated as the sum of eggs laid across the three days and the hatching success was calculated for each pair as the mean daily hatching percentage across the three-day period. Interestingly only around 50% of pairs created both for acetone and control treatment produced (fertilised) eggs. Hence, reproductive measurements were available for four control and five acetone-treated pairs.

Activity was analysed using a negative binomial generalized linear model including treatment, sex, and their interaction. Acetone-versus-Control contrasts were estimated separately for females and males. Because feeding did not differ detectably between sexes, female and male feeding observations were pooled and analysed with treatment as the sole predictor. Total egg production was analysed using a negative binomial generalized linear model with treatment as predictor, whereas mean hatching success was analysed using a linear model with treatment as predictor. All analyses were conducted in R (4).

2.2. Results

Acetone exposure did not significantly affect locomotor activity in either sex. Mean female activity was 20.9 in controls and 17.4 in acetone-treated beetles ($P = 0.758$), whereas mean male activity was 2.1 in controls and 3.2 in acetone-treated beetles ($P = 0.506$; Fig. S2).

Leaf consumption also did not differ significantly between treatments. Mean consumed leaf area was 244.3 mm² in controls and 209.8 mm² in acetone-treated beetles ($P = 0.641$; Fig. S3).

Similarly, acetone exposure did not significantly affect reproductive performance. Mean hatching success was 91.8% in control females and 91.4% in acetone-treated females ($P = 0.941$), while mean three-day egg production was 79 eggs in controls and 92 eggs in acetone-treated females ($P = 0.517$; Fig. S4).

Thus, this additional experiment provided no evidence that acetone exposure alone altered adult locomotor activity, feeding behaviour, egg production, or hatching success under the conditions of the experiment.

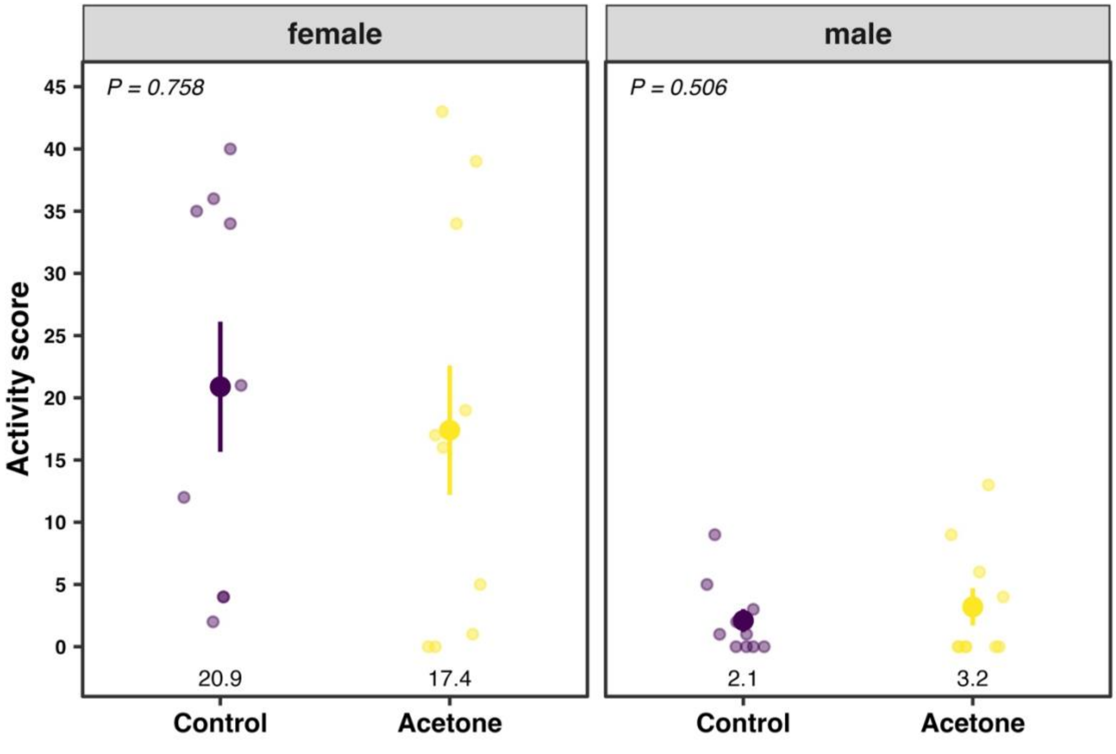

Figure S2. Locomotor activity of adult Colorado potato beetles in the acetone-control experiment. Activity scores of female (left) and male (right) beetles exposed to acetone or left untreated as controls are shown. Points represent individual beetles, and larger points with error bars indicate means ± SE. Numbers above the x-axis indicate group means.

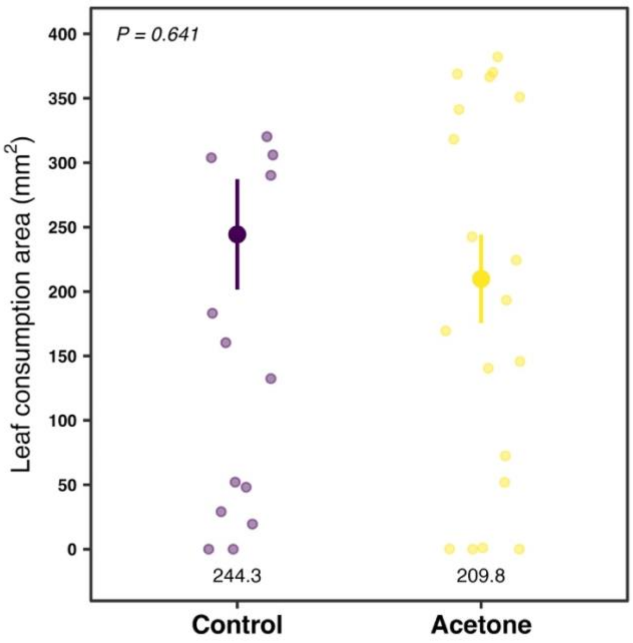

Figure S3. Leaf consumption by adult Colorado potato beetles in the acetone-control experiment. Points represent individual beetles, and larger points with error bars indicate means ± SE. Numbers above the x-axis indicate group means.

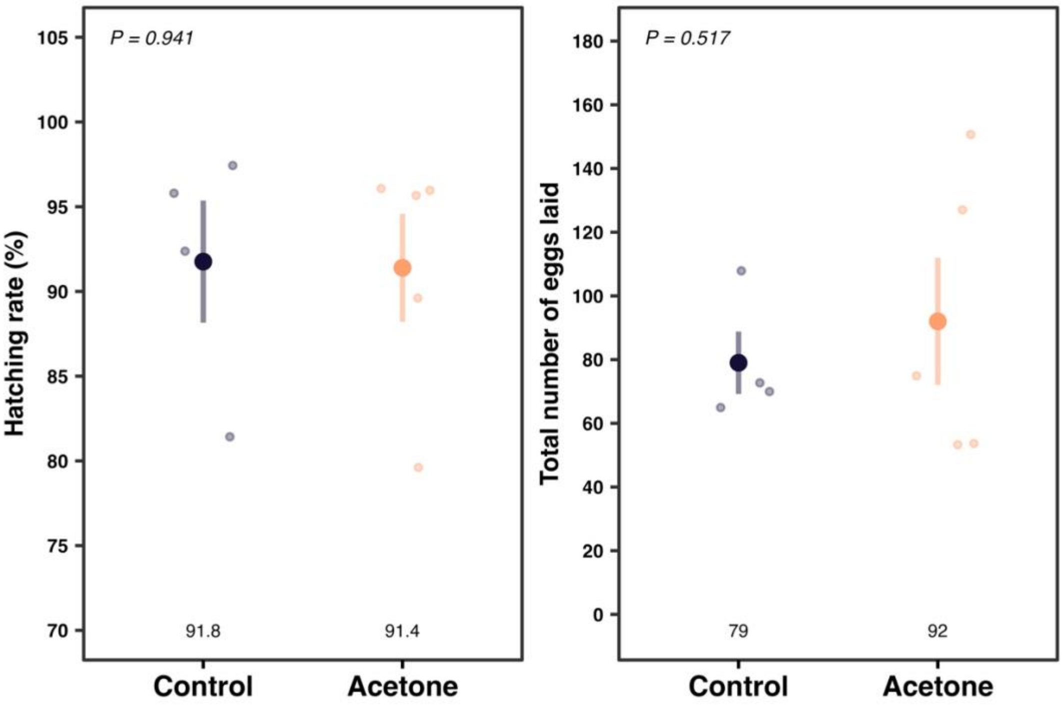

Figure S4. Reproductive performance of Colorado potato beetle pairs in the acetone-control experiment. The left panel shows mean hatching success of eggs produced over three consecutive days, and the right panel shows the total number of eggs laid during this period. Points represent individual pairs, and larger points with error bars indicate means ± SE. Numbers above the x-axis indicate group means.

References

1. He Q, Zhao H. 2021 Imidacloprid in several aqueous co-solvent mixtures: solubility, solvent effect, solvation thermodynamics and enthalpy-entropy compensation. J. Mol. Liq. 338, 116781. (doi:10.1016/j.molliq.2021.116781)
2. Kong MZ, Shi XH, Cao YC, Zhou CR. 2008 Solubility of imidacloprid in different solvents. J. Chem. Eng. Data 53, 615–618. (doi:10.1021/je7004038)
3. Jatsch AS, Ruther J. 2021 Acetone application for administration of bioactive substances has no negative effects on longevity, fitness, and sexual communication in a parasitic wasp. PLoS ONE 16, e0245698. (doi:10.1371/journal.pone.0245698)
4. R Core Team. 2025 R: a language and environment for statistical computing, version 4.5.2. Vienna, Austria: R Foundation for Statistical Computing. See https://www.R-project.org/
